# A case of periodic abdominal pain and fever due to left ovarian abscess

**DOI:** 10.1016/j.radcr.2023.12.056

**Published:** 2024-01-15

**Authors:** Shohei Tanabe, Eriko Uehara, Kotaro Ichida, Takatsugu Kan, Syuji Morishima

**Affiliations:** Kobe City Medical Center, West Hospital, Kobe-shi, Hyogo, Japan

**Keywords:** Ovarian abscess, Laparotomy, Inflammatory disease, Ovarian tumor, Residual abscess

## Abstract

Tubo-ovarian abscess (TOA) is a late complication of pelvic inflammatory disease. Its diagnosis is difficult because it is occasionally accompanied by atypical symptoms such as abdominal pain and fever. A 45-year-old married woman presented with recurrent abdominal pain and fever. Her medical history included ovarian surgery 14 years prior to presentation. Computed tomography (CT) performed by her local doctor confirmed uterine fibroids and a left ovarian tumor. Following a detailed examination and magnetic resonance imaging at our hospital, a TOA was suspected, and surgery was planned. During surgery, the adhesion was firm and required laparotomy. Ultimately, the left ovarian tumor was confirmed to be a TOA. Although complete surgical resection was not feasible. A surgical drain was inserted, and the pus was drained. Cultures revealed *Citrobacter freundii* and other organisms, and oral quinolone antibiotics were administered. CT performed on the fourth postoperative day demonstrated a residual abscess; however, 5 weeks after surgery, CT showed complete resolution of the residual abscess. Subsequently, the antibiotic regimen was terminated, and progestins were administered for the treatment of endometriosis, which is still ongoing.

## Introduction

Tubo-ovarian abscess (TOA) is a late complication of pelvic inflammatory disease (PID) that results in abscesses. Most patients are sexually active, and the risk factors are similar to those of PID [1]. Besides symptoms of abdominal pain and fever, a TOA is difficult to diagnose because of its atypical clinical presentation [2]. In this study, we encountered a case in which a woman with no risk factors for PID, such as a sexually active state or intrauterine manipulation, had recurrent abdominal pain and fever, which made the differential diagnosis difficult. After a thorough examination, we suspected a TOA by magnetic resonance imaging (MRI), and the condition was managed surgically.

## Case presentation

**Fig. 1:**
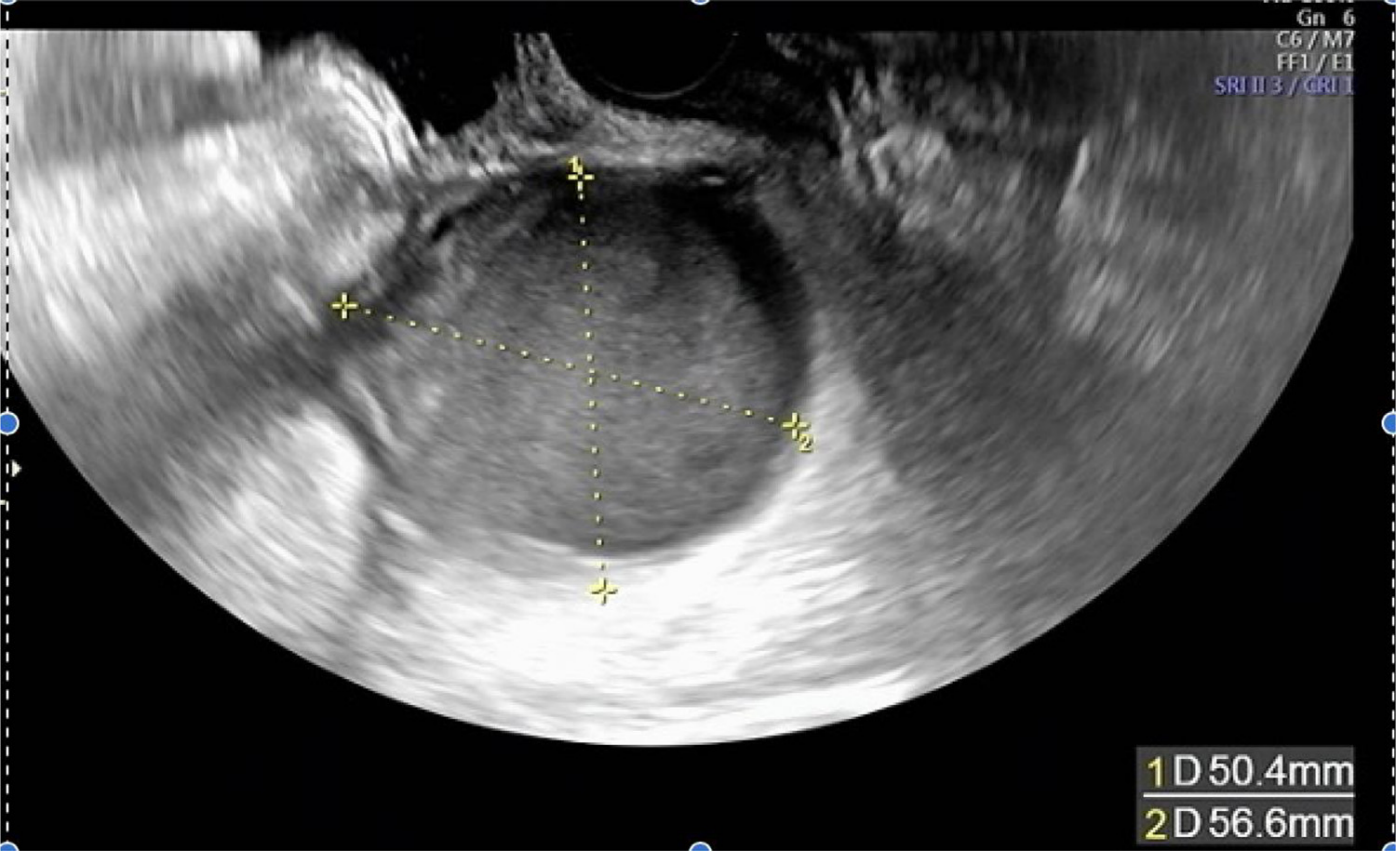
Transvaginal ultrasound showing a 5-cm ovarian tumor (indicated by the yellow lines).

**Fig. 2:**
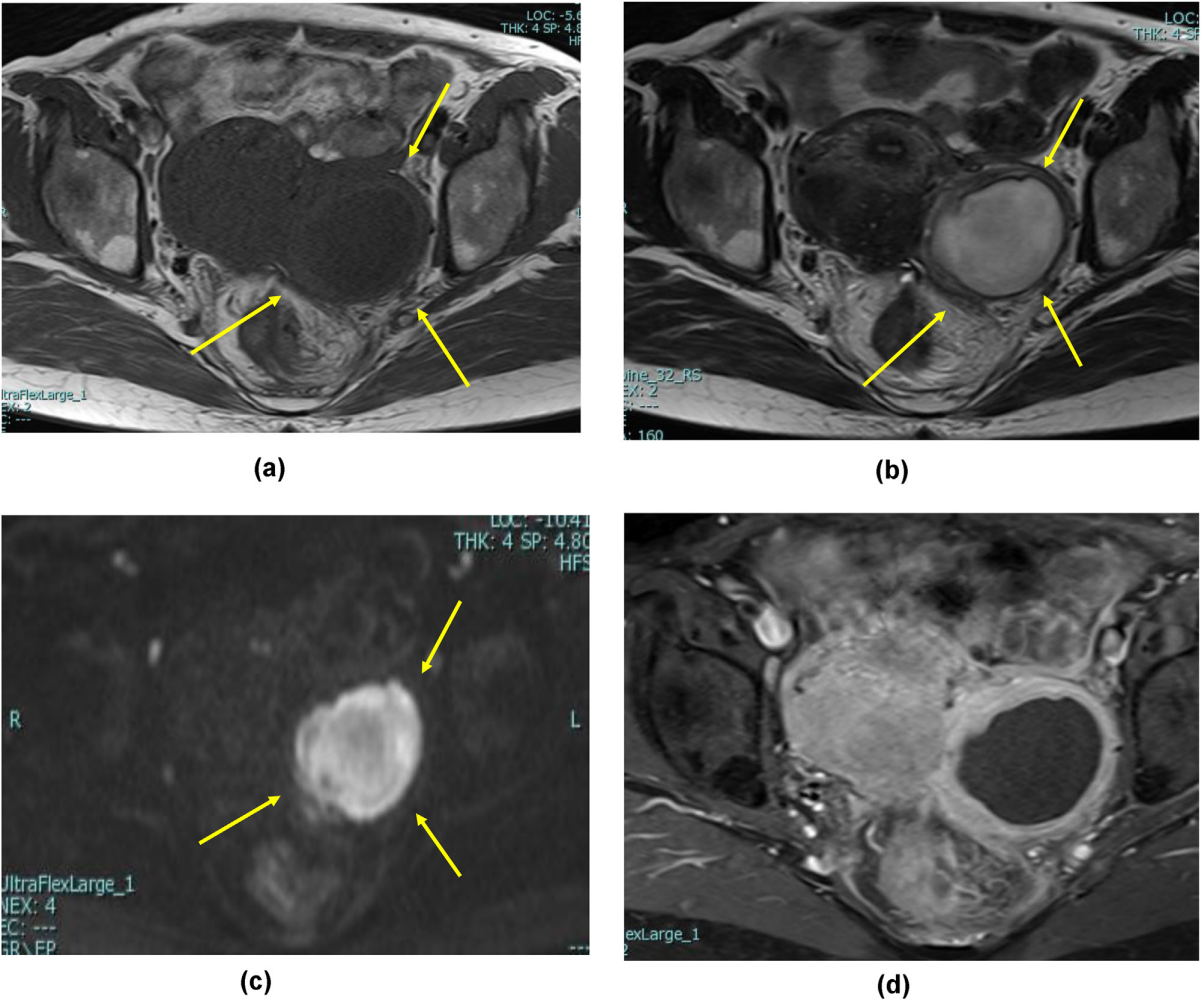
MRI findings of ovarian abscess. Yellow arrow indicates the location of the left ovarian tumor (A) T1-weighted image showing a pale wall and high signal intensity (B) T2-weighted image showing high signal intensity (C) Diffusion-weighted image showing high signal intensity and (D) The abscess wall appears enhanced by the gadolinium contrast.

**Fig. 3:**
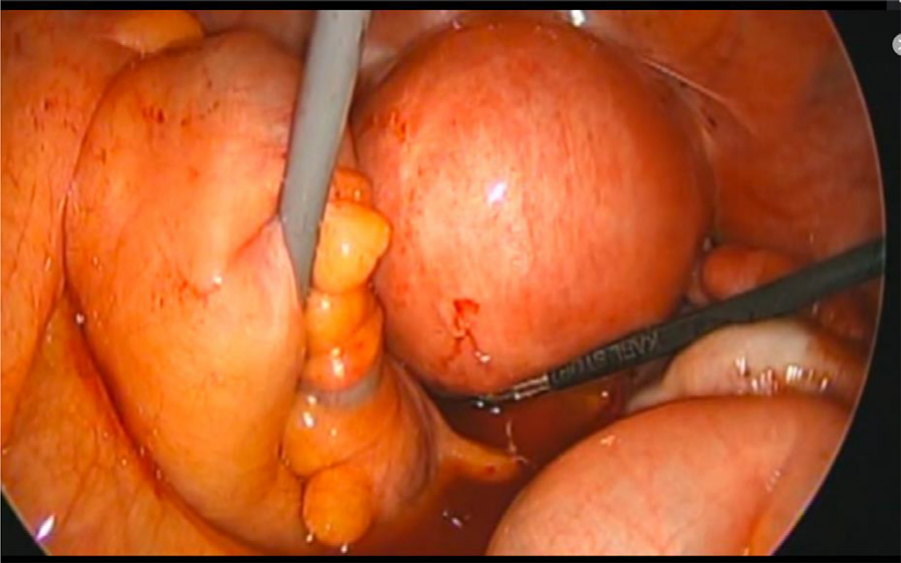
Laparoscopy image showing the left ovarian tumor obscured by the colon and retroperitoneum.

**Fig. 4:**
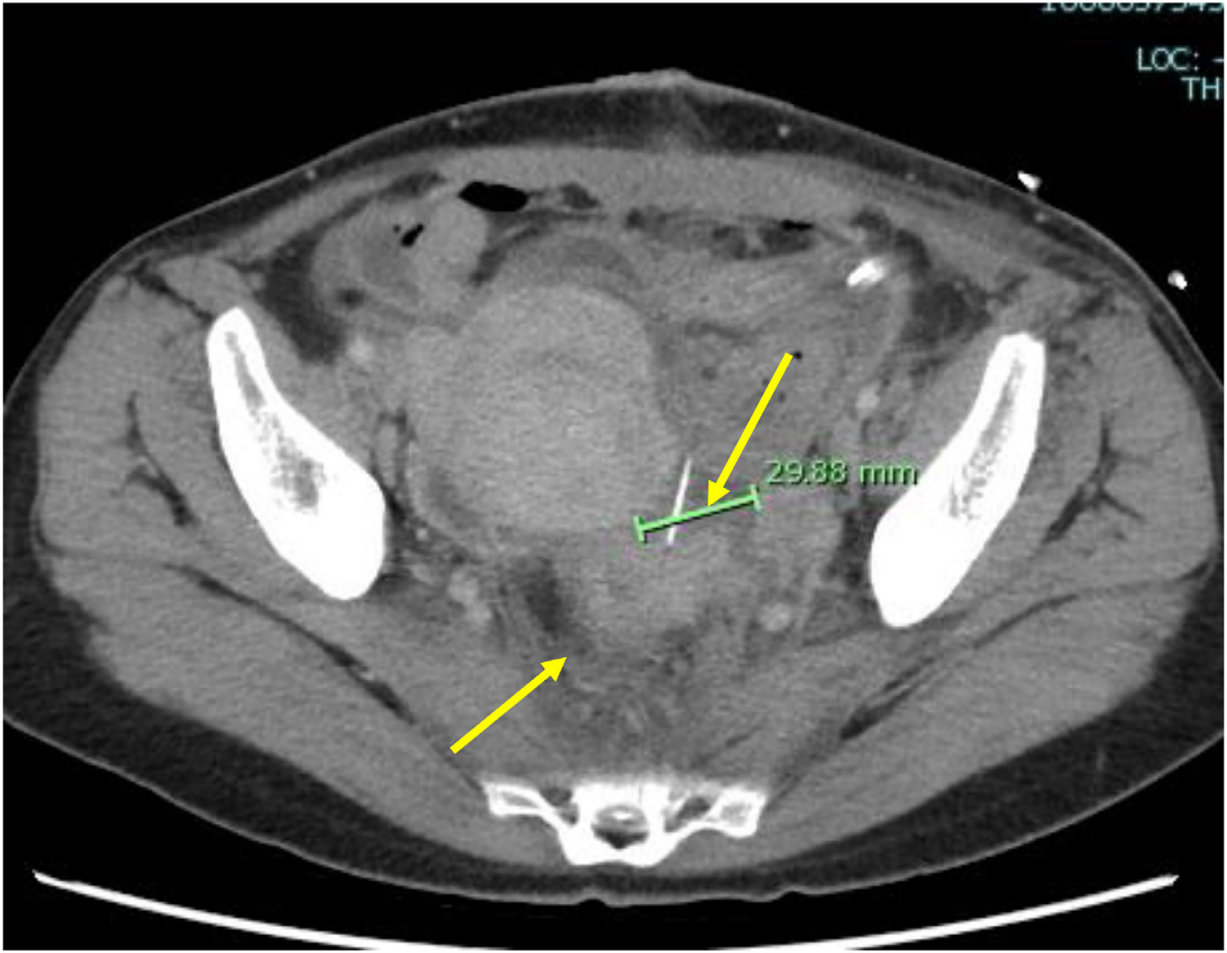
CT performed on the fourth postoperative date: yellow arrow indicating the abscess and surgical drain.

The patient was a 45-year-old married woman with no history of pregnancy. She had undergone laparoscopic ovarian surgery for a left endometrioid cyst at 31 years of age. She had a history of abdominal pain and fever of unrecognized origin for 1 month at the age of 29 and 31 years, respectively. She had abdominal pain and fever for which she visited her primary care physician, who performed computed tomography (CT); based on the imaging findings, diagnoses of uterine myoma, and a left ovarian tumor were made. After the administration of antibiotics, she visited our obstetrics and gynecology clinic for a thorough examination. Upon examination, she was found to have intense tenderness localized in the left lower abdomen. Transvaginal ultrasonography revealed a 5-cm large monovaginal left ovarian tumor (Fig. 1). The pain was so severe that she could not be examined transvaginally for chlamydia or gonorrhea. The same evening, she experienced abdominal pain and fever and visited our emergency room. Pelvic inflammation was suspected, the patient was hospitalized, and cefmetazole 6 g/d with minomycin 200 mg/d were administered. The blood test results at the time of the visit are shown in Table 1. Blood cultures were negative, and on the eighth day, the patient was discharged after completion of antibiotic therapy. Based on the patient's medical history, the physician considered the possibility of collagen disease and performed blood tests for autoantibodies, all of which were negative. The left ovarian tumor was a single ovarian tumor with MRI findings of high signal on T2WI and Diffusion-weighted imaging and pale high signal along the wall on T1W1 and gadolinium contrast (Fig. 2). On imaging, the left ovarian tumor was suspected to be an abscess, although there were no risk factors for ovarian infection, as the patient had no history of sexually transmitted diseases or recent sexual intercourse. The decision was made to perform surgery to determine whether the left ovarian tumor was an abscess and, if so, to drain it. Review laparoscopy revealed that the descending colon firmly adhered to the retroperitoneum. No abscesses were observed in the abdominal cavity. The left ovarian tumor was obscured by the dorsal colon, and the uterus could not be visualized (Fig. 3). Adhesion dissection was initiated laparoscopically; however, the adhesions were strong. Because of intestinal injury, laparoscopic manipulation became difficult, and the abdomen was opened. The pelvic cavity was opened; however, it was impossible to remove the left ovarian tumor because of its strong adhesion to the colon and retroperitoneum. The surgeon performed bowel repair and then repeated adhesion dissection, which resulted in an abscess leaking from within the left ovarian tumor. The left ovarian tumor was thus diagnosed as an abscess; however, abscess removal was not feasible. Hence, the abscess was drained as much as possible and washed with a large volume of saline solution. The surgery was terminated by placing a drain in the fossa of Douglas and within the abscess. CT performed on the same day revealed a 3-cm residual abscess (Fig. 4). Small amounts of *Candida freundii, Bacillus* spp., and *Clostridium* spp. were identified intraoperatively in the abscess. *Neisseria gonorrhoeae* and *Chlamydia* were not detected. Quinolone antibiotics were administered after surgery. Treatment with cefmetazole was initiated after surgery but was discontinued on the third day due to diarrhea. On the seventh postoperative day, a blood test confirmed that the inflammatory response had improved, and the patient was discharged on the eighth day. After 5 weeks of oral quinolone antibiotic therapy, the residual abscess had completely resolved. Thereafter, progestins were administered and continued to the present date for the management of endometriosis.

**Table 1:** Blood test results.

Laboratory parameter	Value	Reference range
White blood cell (/μL)	14,730	3900-9800
Hemoglobin (g/dL)	11.4	11.1-15.1
Platelet (/μL)	314,000	130,000-370,000
Sodium (mmol/L)	143	137-144
Potassium (mmol/L)	3.8	3.6-4.8
Creatinine (mg/dL)	0.72	0.47-0.79
Blood urea nitrogen (mg/dL)	5	6.0-22
CRP (mg/dL)	24.49	<0.3

## Discussion

In the present case, it was difficult to identify a TOA as the cause for recurrent fever and abdominal pain. The presentation of pain and fever was considered atypical as the patient had no history of sexual activity or intrauterine manipulation, which are risk factors for PID. However, the patient had a history of endometriosis, which increases the risk of bacterial vaginosis and PID owing to menstrual blood retention [4].

The differential diagnoses of ovarian abscesses encompass conditions such as endometrioid cysts, appendiceal masses, diverticulitis, and malignancy [2,3]. In this particular case, ovarian tumor was suspected by CT and transvaginal ultrasound, and there were no indications of any other gastrointestinal diseases. Subsequent MRI raised suspicions of an abscess, leading to the diagnosis of an ovarian abscess. The diagnosis was confirmed during the surgery intervention. Notably, the patient's history did not provide the initial indications for suspecting an ovarian abscess.

In the present case, the atypical signs and symptoms made the diagnosis of an ovarian abscess challenging; however, MRI proved to be a valuable tool. For the detection of an ovarian abscess, MRI has a sensitivity of 83.3% compared with that of transvaginal ultrasonography (58.3%) and has been reported to be equally useful [5]. MRI may be used to differentiate ovarian tumors from abscesses when transvaginal ultrasonography renders indefinite diagnosis.

Ovarian abscess treatment can be divided into antibiotic and surgical therapy. It has been reported that antibiotic treatment has a high risk of failure in patients aged >40 years with fever and large abscesses [6]. Age and fever were relevant to the present case. Furthermore, no correlation has been established between the diameter of the abscess and risk of antibiotic treatment failure [6], [7], [8]. Therefore, it would be safer to use age and fever as criteria for judgment, rather than size alone.

## Conclusion

Women without common PID risk factors may develop a TOA if they have a history of endometriosis, and MRI may provide useful information for the diagnosis and treatment planning for a TOA.

## Author contributions

Shohei Tanabe wrote the manuscript draft. Eriko Uehara, Kotaro Ichida, Takatsugu Kan, and Syuji Morishima reviewed and revised the draft critically for intellectual content. All authors contributed to the manuscript draft and have approved the final version of the manuscript.

## Ethical statement

This study was approved by our hospital's Clinical Research Review Committee.

## Patient consent

Written informed consent was obtained from the participant before the execution of any study-related procedures.
